# Multiomics reveals changes in lipid metabolism in the livers of Landes geese before and after overfeeding

**DOI:** 10.5713/ab.25.0405

**Published:** 2025-08-12

**Authors:** Weiqing Ma, Liu Yang, Yadi Jing, Pengwei Ren, Xiang Liu, Meixia Zhang, Xiaomin Qi, Mingxia Zhu, Qiaomei Zhang

**Affiliations:** 1 College of Agriculture and Biology, Liaocheng University, Liaocheng, China; 2 Shandong Jinxin Lande Goose Industry Co., Ltd., Jining, China; 3 Academic Affairs Office, Liaocheng University, Liaocheng, China

**Keywords:** Foie Gras, Geese, Metabolome, Microbiome, Overfeeding

## Abstract

**Objective:**

The aim of this experiment was to integrate production indices with omics sequencing to elucidate the systemic perturbations between hepatic metabolism and the gut microbiota during overfeeding.

**Methods:**

A total of 120 seven-week-old male Landes geese were floor-reared in a pen environment. Overfeeding commenced at week 8 using a corn-based diet containing 5% soybean oil. The feeding regimen consisted of three daily meals (150–180 g/meal) initially, gradually increasing to five meals (300–500 g/meal) after two weeks, and was maintained for a total overfeeding period of four weeks.

**Results:**

The results demonstrated that overfeeding significantly increased liver weight and serum lipid levels, accompanied by intracellular lipid droplet accumulation. Concurrently, the downregulation of taurine-conjugated bile acids and the upregulation of free bile acids disrupted cholesterol homeostasis. Crucially, overfeeding triggered gut microbial dysbiosis characterized by *Escherichia–Shigella* enrichment and *norank_o_Clostridia_UCG-014* depletion.

**Conclusion:**

Our work demonstrated that the identification of the “gut microbiota-bile acid-liver axis” could serve as a pivotal signaling pathway driving overfeeding-induced foie gras formation while providing a theoretical foundation for overfeeding strategies to mitigate metabolic pathologies in waterfowl production.

## INTRODUCTION

Foie gras is considered one of the representatives of high-grade ingredients, characterized by its melt-in-the-mouth texture and delicate meat quality. It is nutritionally rich in polyunsaturated fatty acids, which help soften blood vessels, offering beneficial health effects for cardiovascular and cerebrovascular diseases [1]. Generally, the Landes goose, as a large breed, has excellent foie gras production performance. The foie gras it produces weighs between 700 and 900 g, making it a top-grade product. During production, 8-week-old Landes geese are typically fed high-calorie feed for 3 to 4 weeks to produce top-grade foie gras products. Due to the relatively long overfeeding period, the production of foie gras is significantly limited, which restricts the development of the foie gras industry.

During migration, migratory birds tend to accumulate a substantial amount of food in advance. The excess energy is deposited in the liver, resulting in fatty liver. The liver is the primary organ for lipid synthesis in poultry. Overfeeding can increase the liver’s capacity to synthesize triglycerides (TG) beyond the rate of lipid transport and breakdown, thereby leading to the accumulation of lipid droplets and an enlarged liver [2,3]. Following the expenditure of energy, the liver reverts to its previous normal state, making the entire process reversible [4]. However, unlike those of alcoholic liver disease (ALD) and nonalcoholic fatty liver disease (NAFLD), the livers of geese rarely exhibit significant pathological changes. At present, research on the mechanism of foie gras formation has focused mainly on lipid metabolism and other directions. In a study involving the induction of foie gras production in lion-headed geese through overfeeding, transcriptomics and lipidomics revealed that the formation of lion-headed foie gras led to the activation of key signalling pathways, such as the biosynthesis of unsaturated fatty acids, glycerolipid metabolism, and glycerophospholipid metabolism. These analyses identified several critical genes, including 1-acylglycerol-3-phosphate O-acyltransferase 2 (*AGPAT2*), diacylglycerol O-acyltransferase 2 (*DGAT2*), phospholipase D family member 4 (*PLD4*), and phospholipase A2 group IVF (*PLA2G4F*) [5]. The supplementation of essential oils can upregulate the mRNA expression levels of genes involved in fat production, such as acetyl-CoA carboxylase α (*ACCα*), carbohydrate response element-binding protein (*ChREBP*), and sterol regulatory element-binding protein 1 (*SREBP-1*), while downregulating the mRNA expression levels of all genes involved in fatty acid oxidation [6]. These findings indicate that the production of foie gras is not only related to the synthesis of fatty acids in the liver but also influenced by the catabolism of fat. In addition, the regulation of lipid metabolism by microorganisms and their metabolites plays an important role in the production of foie gras.

In recent years, with the development of microbiome technologies, the association between the gut microbiota and visceral organs has become increasingly tight. Studies have shown that the gut microbiota can drive the reprogramming of hepatic transcriptional metabolism [7]. Research has shown that an imbalance in the gut microbiota can affect anti-tumour immune surveillance and promote the progression of liver disease to cancer [8]. Therefore, the composition and diversity of the gut microbiota have become a focus of research on liver lipid metabolism. Excessive intake of dietary fructose in overweight/obese adolescents was associated with a decrease in *Eubacteria* and *Streptococcus*, which are involved in carbohydrate metabolism in the gut microbiota [9]. The combined analysis of plasma and liver metabolomics revealed that the upregulation and enrichment of glycerolipid metabolites in high-fat diet (HFD)-fed hamsters were positively correlated with *Faecalibaculum*, *Allobacterium*, and *Eubacterium generata*, as well as plasma glycerolipid metabolites and lipid indices [10]. Similarly, microorganisms also mediate the occurrence of liver metabolic diseases. A HFD significantly increases the activity of bile salt hydrolase (BSH) in the mouse ileum microbiota, leading to an increase in the content of free bile acids in the ileum. This, in turn, inhibits the synthesis of bile acids in the liver, damaging lipid metabolism and homeostasis [11]. During the progression of NAFLD, microbial diversity significantly decreases and is significantly correlated with increased levels of LPS biosynthesis, tryptophan metabolism, glutathione metabolism, and lipid metabolism [12]. Numerous studies on dietary protection against NAFLD have shown that the gut microbiota plays an important role in this process. Sulforaphane protected the intestinal integrity of mice induced by a high-fructose/high-fat acid diet and reduced the transport of gut-derived LPS to the liver, weakening NAFLD through the gut-liver axis [13]. *L*-Arabinose can promote hydrogen release and alleviate HFD-induced liver steatosis, which can be eliminated by antibiotic treatment, confirming the regulatory role of the gut microbiota in liver steatosis [14]. Although it has been reported that the abundance of *Ruminococcus gnavus* is significantly enriched in individuals who are overweight or obese and is positively correlated with serum total cholesterol (TC) and hepatic fat, this finding does not imply that the progression of fatty liver disease is influenced by a single microbial strain or factor [15]. Therefore, the use of combined multiomics analysis is one of the hot research directions for studying liver lipid metabolism.

An increasing number of studies have shown the involvement of microorganisms in lipid metabolism in ALD and NAFLD. However, the relationship between lipid metabolism in the liver of Lande geese and the gut microbiota has not been elucidated. Therefore, this study analyses the mechanism of foie gras production from the perspective of the gut-liver axis, providing new insights for studying the formation mechanism of foie gras.

## MATERIALS AND METHODS

### Goose feeding and management

A total of 120 seven-week-old male Landes geese were floor-reared in a pen environment. Overfeeding commenced at week 8 using a corn-based diet containing 5% soybean oil. The feeding regimen consisted of three daily meals (150–180 g/meal) initially, gradually increasing to five meals (300–500 g/meal) after two weeks, and was maintained for a total overfeeding period of four weeks. The experimental design included two sampling phases: before overfeeding (BO) at week 7 and after overfeeding (AO) post completion of the force-feeding period. Eight birds per phase were fasted for 12 hours (the day before BO sampling and after AO treatment completion) prior to blood collection from the wing vein and subsequent slaughter. This experiment was approved by the Animal Ethics Committee of Liaocheng University (AP2024061217) and conducted under the guidance of the committee.

### Sample collection

After the serum was collected through the inferior vena cava, it was allowed to stand for 1 hour and then centrifuged at 3,000 r/min for 15 minutes. A pipette was used to aspirate the serum into a 1.5 mL EP tube, which was subsequently stored at 4°C for testing. After dissection, the goose’s abdominal fat, liver, and caecal chyme were removed. The abdominal fat and liver were weighed, and the abdominal fat index (AFI) and liver index (LI) were calculated. A portion of the liver was placed in a centrifuge tube containing 4% paraformaldehyde for detecting liver tissue morphology. The other portions of the liver and caecal chyme were divided into cryovials and quickly frozen in liquid nitrogen before being transferred to a −80°C freezer for subsequent determination of liver metabolomics and the caecal chyme microbiome.

### Serum biochemistry

Serum TC, triglycerides (TG), low-density lipoprotein (LDL), and high-density lipoprotein (HDL) levels were quantified using a fully automated biochemical analyser (Mindray BS-800 M; Shenzhen Mindray Biomedical Electronics).

### Liver tissue analysis

The main steps of haematoxylin and eosin (HE) staining are as follows: remove the liver tissue soaked in fixative; dehydrate it with alcohol, alcohol benzene, and xylene; immerse it in wax in sequence; embed it in a tissue embedding machine; and cool it in a −20°C freezer. After cooling, the wax block was placed in a paraffin slicer to cut 4 μm thick slices, which were subsequently dried in a 60°C oven, stained with HE, and finally sealed with dehydrated neutral gum. The main steps of oil red O staining are as follows: replace the liver fixative with a 15% sucrose solution at 4°C overnight, replace the 15% sucrose solution with a 30% sucrose solution at 4°C overnight the next day, cover the liver tissue with OCT embedding agent, and place it in a frozen slicer until completely solidified for sectioning. The frozen slices were frozen and dried, oil red O working solution was added, the samples were counterstained with hematoxylin, and finally, the slices were sealed with glycerol gelatine. Both HE staining and oil red O staining were performed using a high-resolution slide scanning system Pannoramic MIDI (3DHISTECH) to collect images.

### Liver metabolome

The samples stored at −80°C were thawed on ice and homogenized using a grinding mill (30 Hz) for 20 s. A 20 mg aliquot of the homogenate was mixed with 400 μL of internal standard extraction solution (methanol:water = 7:3, v/v) and vortexed at 1,500 rpm for 5 min. After 15 min of incubation on ice, the mixture was centrifuged at 11,304×g for 10 min at 4°C, and 300 μL of the supernatant was collected. The supernatant was stored at −20°C for 30 min, followed by a second centrifugation at 11,304×g for 3 min at 4°C. A 200 μL aliquot of the resulting supernatant was subjected to liquid chromatography-mass spectrometry (LC-MS) analysis. Chromatographic separation was performed on a Waters ACQUITY Premier HSS T3 column (1.8 μm, 2.1 mm×100 mm; Waters, MA, USA) with a mobile phase consisting of 0.1% formic acid in water (phase A) and 0.1% formic acid in acetonitrile (phase B). The gradient elution program was as follows: 0.0–2.0 min, 95% A to 80% A; 2.0–5.0 min, 80% A to 40% A; 5.0–6.0 min, 40% A to 1% A; 6.0–7.5 min, 1% A; 7.5–7.6 min, 1% A to 95% A; and 7.6–10.0 min, 95% A. The column temperature was maintained at 40°C, with a flow rate of 0.4 mL/min and an injection volume of 4 μL. Mass spectrometry was conducted using a Thermo Scientific Q Exactive HF-X instrument (Thermo Fisher Scientific) equipped with electrospray ionization (ESI) in positive/negative switching mode. Full-scan spectra were acquired over a mass range of m/z 75–1,000 with resolutions of 35,000 (MS1) and 17,500 (MS2). The ion source parameters included spray voltages of 3,500 V (positive) and 3,200 V (negative), a sheath gas flow rate of 30 Arb, an auxiliary gas flow rate of 5 Arb, an ion transfer tube temperature of 320°C, and a vaporizer temperature of 300°C. Data acquisition alternated between full-scan and data-dependent MSn modes, with dynamic exclusion to minimize redundant scans, ensuring efficient metabolite separation and identification.

### Intestinal chyme microbiome

Microbiome were taken from caecal chyme. The E.Z-N. A soil DNA kit (Omega Bio tek) was used to extract the total genomic DNA of the intestinal chyme microbiota. Using the extracted DNA as a template, PCR amplification was performed on the V3–V4 variable region of the 16S rRNA gene using the upstream primer 338F (5′-ACTCCTACGGGAGGCAGCAG -3′) and the downstream primer 806R (5′-GACTACHVGGG TWTCTAAT-3′) carrying the barcode sequence. The PCR system consisted of 4 μL of TransStart FastPfu buffer, 2 μL of 2.5 mM dNTPs, 0.8 μL of upstream primer (5 μM), and 0.8 μL of downstream primer (5 μM). The FastPfu DNA polymerase (0.4 μL) and template DNA (10 ng) were transformed, and 20 μL was added. The amplification program of the PCR instrument (ABI 9700; Thermo Fisher Scientific) involved predenaturation at 95°C for 3 minutes, 27 cycles (denaturation at 95°C for 30 s, annealing at 55°C for 30 s, and extension at 72°C for 30 s), a stable extension at 72°C for 10 minutes, and storage at 4°C. The NEXTFLEX Rapid DNA Seq Kit was used to construct a library of purified PCR products. Sequencing was performed via the Illumina NextSeq 2000 platform (Illumina).

### Data analysis

Statistical analyses and graphical visualizations were conducted using GraphPad Prism (GraphPad Software). Independent samples t tests were applied for group comparisons, and Pearson correlation analysis was used to assess variable associations. Wilxocon rank sum test was used to analyze the intergroup differences in alpha diversity. Principal coordinate analysis (PCoA analysis) based on the Bray Curtis distance algorithm was used to test the similarity of microbial community structure between samples, and PERMANOVA non parametric test was used to analyze whether the differences in microbial community structure between sample groups were significant.

## RESULTS

### Overfeeding causes visceral fat deposition

**Figure 1:**
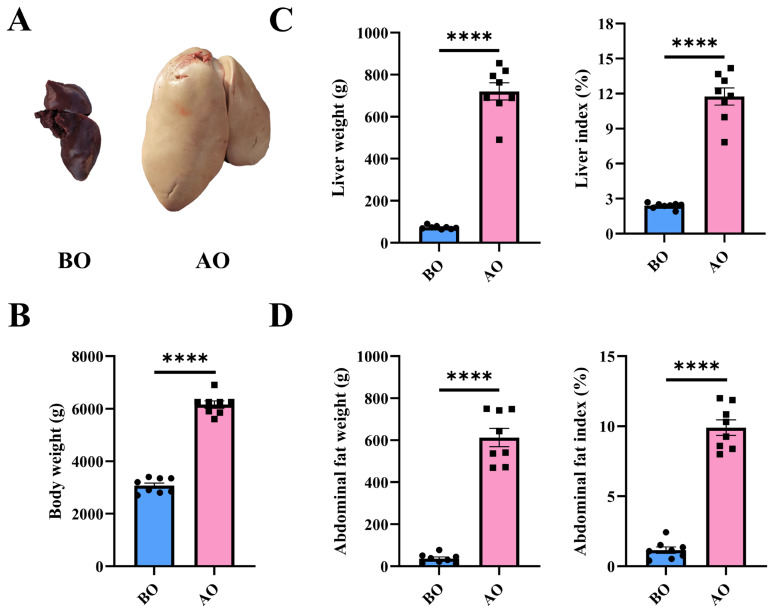
Changes in the liver and abdominal fat before and after overfeeding. (A) Morphology of the liver before and after overfeeding. (B) Body weights of Landes geese before and after overfeeding. (C) Liver weight and liver index before and after overfeeding. (D) Abdominal fat weight and index before and after overfeeding. n = 8. Data are presented by average±SEM. **** indicates p<0.0001. BO, before overfeeding; AO, after overfeeding; SEM, standard error of the mean.

To investigate the changes in the liver of Landes geese AO, we recorded the body weight before slaughter and the weights of the liver and abdominal fat after slaughter. The results revealed that overfeeding led to an enlargement of the liver, with its colour changing from reddish-brown to yellowish-white (Figure 1A). Similarly, the body weight, liver weight (LW), LI, abdominal fat weight, and AFI of the Landes geese all significantly increased (Figures 1B–1D).

### Overfeeding increases lipid metabolism

**Figure 2:**
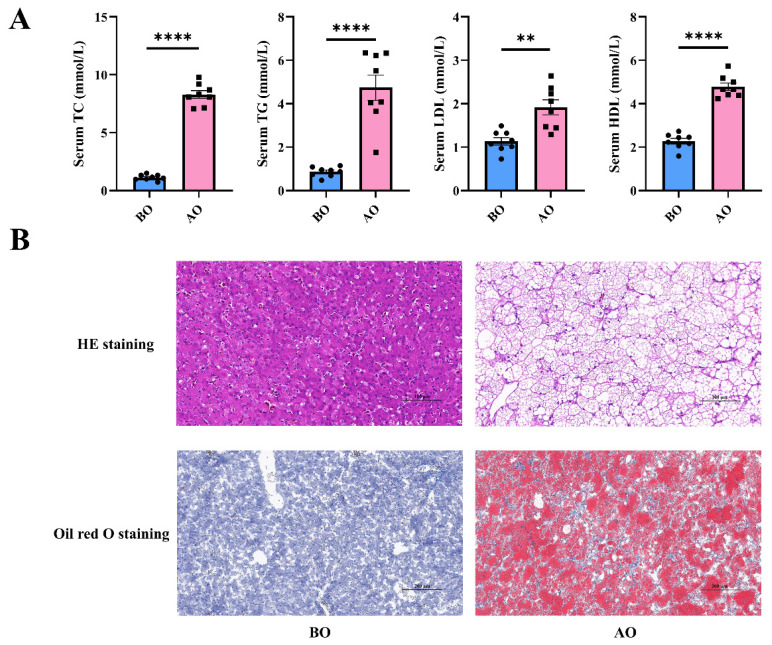
Serum lipid metabolism and liver tissue staining before and after overfeeding. (A) Serum TC, TG, LDL and HDL levels, n = 8. Data are presented by average±SEM. ** indicates p<0.01, **** indicates p<0.0001. (B) Liver HE staining and oil red O staining, with scale bars of 100 μm and 200 μm, respectively. BO, before overfeeding; AO, after overfeeding; LDL, low-density lipoprotein; HDL, high-density lipoprotein; SEM, standard error of the mean; HE, haematoxylin and eosin.

To further investigate the effects of overfeeding on lipid metabolism in Landes geese, we measured serum lipid metabolism-related indicators. The results revealed that overfeeding significantly increased the levels of serum TG, TC, LDL, and HDL (Figure 2A). Histological staining with HE and Oil Red O revealed that overfeeding led to the appearance of vacuoles in liver cells, which were filled with lipid droplets and presented characteristics of hepatic steatosis (Figure 2B).

### Overfeeding alters the liver metabolome

**Figure 3:**
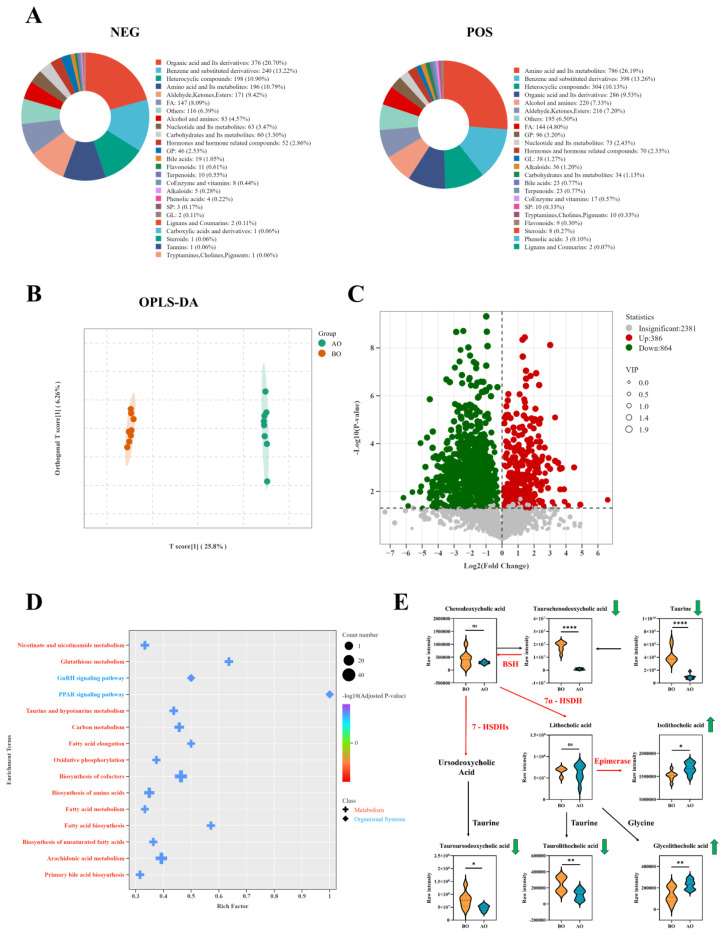
Changes in the liver metabolome before and after overfeeding. (A) Composition of liver metabolites. (B) OPLS-DA score chart. (C) Volcano plot. (D) Differentially abundant metabolite KEGG enrichment analysis. (E) Differentially abundant metabolites, n = 8. Data are presented by average±SEM. * indicates p<0.05, ** indicates p<0.01, **** indicates p<0.0001. Red arrows and abbreviations indicate microbial metabolic pathways, while black arrows and abbreviations indicate host metabolic pathways. AO, after overfeeding; BO, before overfeeding; BSH, bilesalthydrolase; 7-HSDHs, 7-Hydroxysteroid dehydrogenases; 7α-HSDH, 7α-Hydroxysteroid dehydrogenase; SEM, standard error of the mean.

As shown in Figure 3A, the principal hepatic metabolites BO and AO were predominantly categorized as organic acids and derivatives, benzene and substituted derivatives, heterocyclic compounds, and amino acids with their metabolites. The OPLS-DA score plot revealed a clear separation between the pre- and post overfeeding groups, indicating substantial metabolic profile differences (Figure 3B). Volcano plot analysis revealed 386 significantly upregulated and 864 significantly downregulated differentially abundant metabolites following overfeeding (Figure 3C). KEGG pathway enrichment analysis of these differentially abundant metabolites revealed predominant involvement in primary bile acid biosynthesis, taurine and hypotaurine metabolism, and the biosynthesis of cofactors (Figure 3D). Notably, key bile acid metabolites, including taurochenodeoxycholic acid (TCDCA), taurine, tauroursodeoxycholic acid, and taurolithocholic acid, were significantly downregulated post overfeeding, whereas isolithocholic acid and glycolithocholic acid were markedly upregulated (Figure 3E).

### Response of the caecal chyme microbiota to overfeeding

**Figure 4:**
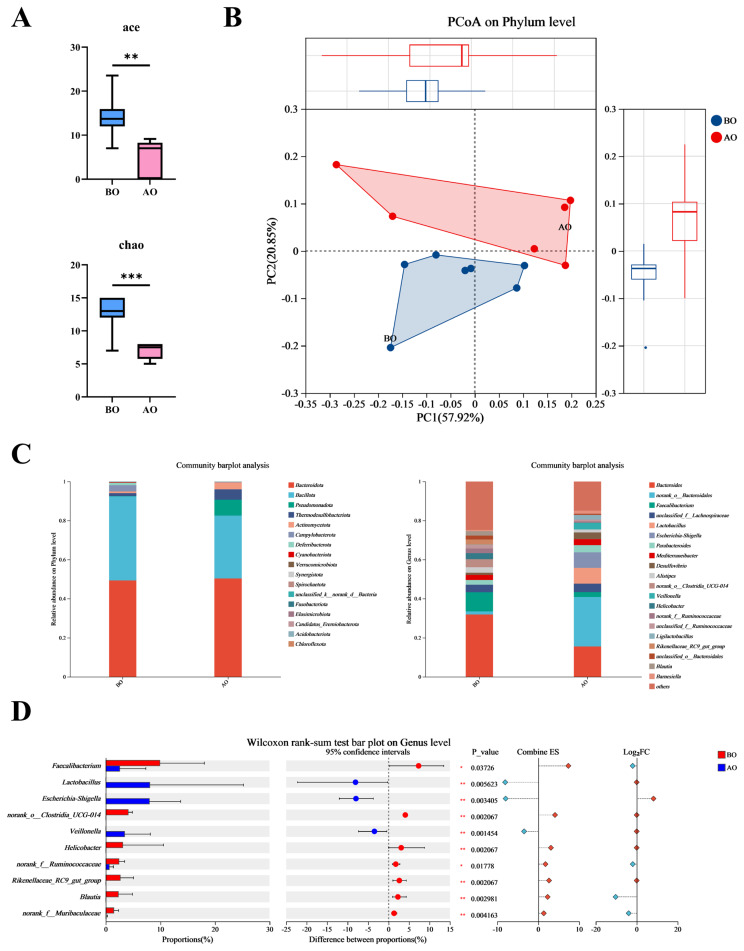
Caecal digesta microbiome. (A) α diversity. Data are presented by average±SEM. ** indicates p<0.01, *** indicates p<0.001. (B) β diversity. (C) Composition of the caecal digesta microbiota. Grouped samples were calculated using the mean, and the top 20 aggregated abundances were taken. (D) Differential microbes in the caecal digesta. Identify differentially abundant microbes at the genus level using the Wilcoxon rank-sum test, with FDR correction for multiple testing. BO, before overfeeding; AO, after overfeeding; SEM, standard error of the mean.

Gut microbes play a key role in crosstalk along the gut-liver axis. To investigate the changes in the composition and abundance of gut microbes AO, we performed 16S rRNA gene sequencing on the caecal digesta microbiota. The results revealed that overfeeding significantly reduced the α-diversity index of the caecal digesta microbiota (Figure 4A). PCoA revealed a clear separation of the caecal digesta microbiota before and AO (Figure 4B). The species composition indicated that the microbial composition at the phylum and genus levels differed in abundance between the two groups AO (Figure 4C). Differential microbe analysis revealed that overfeeding significantly reduced the abundance of certain microbes while increasing the abundance of others (Figure 4D).

### Correlation analysis

**Figure 5:**
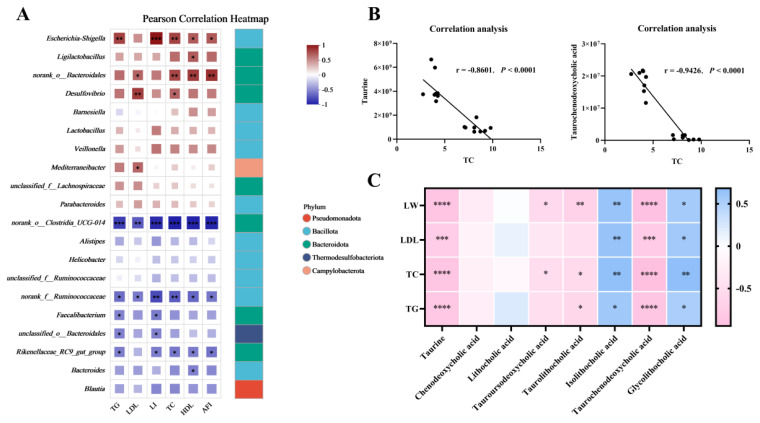
Pearson correlation analyses. (A) Correlations between caecal microbiota and production performance and serum biochemistry. (B) Associations of differentially abundant metabolites with TC. (C) Correlation heatmap of differentially abundant metabolites with production performance and serum biochemistry. LDL, low-density lipoprotein; LI, liver index; HDL, high-density lipoprotein; AFI, abdominal fat index; LW, liver weight.

Further correlation analysis revealed that *Escherichia-Shigella* exhibited significant positive correlations with TC and LI, whereas *norank_o_Clostridia_UCG-014* exhibited significant negative correlations with these parameters (Figure 5A). Analysis of the associations between differentially abundant metabolites and TC revealed that both taurine and TCDCA were significantly negatively correlated with TC, LW, LDL, and TG (Figures 5B, 5C). Finally, source-tracking analysis indicated that taurine and TCDCA were traceable to bacterial origins (Table 1).

## DISCUSSION

Overfeeding is a husbandry practice that rapidly increases body weight when high-energy diets are administered within a short timeframe. Studies have demonstrated that during overfeeding, compared with conventionally fed birds, Lion-Head geese exhibit a 3.5-fold increase in LW [7]. Excessive energy intake during overfeeding directly provides substrates for lipogenesis. As the central organ for lipid metabolism, the liver under high-energy loading activates the sterol regulatory element-binding protein-1c/fatty acid synthase (SREBP-1c/FASN) axis to increase TG synthesis while suppressing fatty acid oxidation, leading to aberrant intracellular lipid droplet accumulation [16,17]. Lipid droplets occupy the cytoplasmic space, displace organelles, and reduce vascular demand, thereby altering organ morphology. Consequently, we observed a shift in liver colour from heme- and cytochrome-rich reddish brown to lipid-dominated pale yellow AO. Furthermore, overfeeding significantly increased both liver and abdominal fat indices, with pronounced abdominal fat deposition indicating systemic metabolic dysregulation beyond hepatic targeting.

The TG, TC, and LDL are key biomarkers of lipid metabolism. High-energy diets increase the number of hepatic lipid droplets, altering metabolites involved in bile acid and lipid metabolism [18]. In metabolic disorders such as NAFLD, TG can play a role in fixing and protecting internal organs in the human body. However, when the accumulation level of TG increases, it reduces the sensitivity of cells to insulin, promotes abnormal glucose and lipid metabolism in the liver, causes liver function damage in patients, and leads to NAFLD [19,20]. Animal experiments have also shown that abnormal lipid metabolism is involved in the increase of liver fat. After excessive feeding, the liver metabolic pathways of Tianfu meat geese primarily focused on glucose and lipid metabolism, amino acid metabolism, the inflammatory response, and the cell cycle. Peripheral adipose tissue and the liver synergistically regulated the accumulation of liver lipids [21]. Integrated transcriptomic and lipidomic analyses have indicated that dietary supplementation with 10% fructose can increase lipid synthesis and cell growth in the liver, thereby improving the production efficiency and quality of foie gras in Tianfu meat geese [22]. Bile acid biosynthesis serves as the primary pathway for endogenous cholesterol metabolism. The hepatic enzymatic conversion of cholesterol to bile acids is regulated by feedback mechanisms to maintain cholesterol homeostasis [23]. LDL transports cholesterol into cells via LDL receptor (LDLR) binding, while HDL mediates reverse cholesterol transport to the liver for biliary excretion [24,25]. Our results revealed elevated serum TG, TC, LDL, and HDL levels in force-fed geese. Notably, increased HDL may reflect compensatory regulation under lipid overload. Histopathological findings further confirmed overfeeding-induced hepatic lipid dysregulation, with vacuolization observed via HE staining and lipid droplet enrichment observed via oil red O staining.

Primary bile acids are synthesized in hepatocytes from cholesterol via rate-limiting enzymes such as cholesterol 7α-hydroxylase (CYP7A1). These acids not only serve as critical mediators of lipid digestion and absorption but also regulate hepatic lipid synthesis and catabolism by activating the farnesoid X receptor (FXR) [26]. Taurine, an essential substrate for the formation of taurine-conjugated bile acids [27], plays a pivotal role in this process. Taurine deficiency may shift hepatic synthesis towards free-form bile acids or glycine-conjugated bile acids. Free bile acids exhibit weaker FXR activation capacity, failing to effectively suppress SREBP-1c-mediated lipogenesis while potentially promoting adipose tissue lipolysis via TGR5 receptor activation, thereby increasing circulating free fatty acid influx to the liver [28,29]. In poultry, glycine-conjugated bile acids demonstrate reduced stability and functionality, impairing enterohepatic recycling efficiency and exacerbating negative feedback inhibition of bile acid synthesis [30]. In this study, overfeeding significantly downregulated taurine and its conjugated bile acids, suggesting that suppressed taurine-conjugated bile acid synthesis leads to hepatic cholesterol accumulation. This disruption interferes with bile acid synthesis and modification pathways, ultimately destabilizing hepatic lipid metabolic homeostasis. These findings provide novel mechanistic insights into overfeeding-induced hepatic steatosis in geese.

Advances in omics technologies have positioned microbiomics as a critical tool for studying host–microbiota metabolic interactions [31]. In lipid metabolism research, Jiang et al [32] demonstrated that biotransformed bile powder modulates lipid metabolism by promoting *Lactobacillus* growth and γ-aminobutyric acid production. Our 16S rRNA sequencing revealed systemic perturbations in the caecal microbiota composition of force-fed Landes geese, characterized by reduced α diversity, increased *Escherichia–Shigella* abundance, and decreased *norank_o_Clostridia_UCG-014* abundance. These findings validate the central role of the “gut microbiota-metabolite-liver” axis in overfeeding-induced hepatic lipid dysregulation.

The potential pathogenic genera *Escherichia-Shigella* were positively correlated with TC and LI. Its proliferation may dissociate conjugated bile acids into free forms via BSH activity, reducing the efficiency of enterohepatic bile acid recycling. This forces the liver to accelerate cholesterol consumption to compensate for bile acid depletion, indirectly stimulating cholesterol synthesis [33]. In contrast, *norank_o_Clostridia_UCG-014* exhibited negative correlations with TC and LI. This taxon has carbohydrate fermentation capacity, producing short-chain fatty acids (SCFAs) that suppress hepatic cholesterol synthesis by downregulating the rate-limiting enzyme *HMGCR* via intestinal FFAR3 receptor activation while upregulating the reverse cholesterol transport proteins *ABCG5/ABCG8* to reduce serum TC levels [34–36]. Traceability analysis further suggested microbial origins for taurine and TCDCA. *Clostridia_UCG-014* likely converts primary bile acids to secondary forms through 7α-dehydroxylation, subsequently activating FXR signalling to inhibit SREBP-1c-mediated lipogenesis, thereby alleviating hepatic steatosis [37]. Lipidomics revealed that taurine can reduce the TG content of 18:2 n6 at the sn-2 and sn-3 positions on the glycerol backbone to decrease lipid accumulation [38]. Meanwhile, this also suggests that we can develop and utilise norank_o_Clostridia-UCG-014 or taurine as probiotics or functional feed additives. Integrative analysis of the gut microbiota composition, metabolomic profiles, and lipid metabolic phenotypes revealed a novel mechanism: overfeeding-induced dysregulation of *Escherichia–Shigella* and *norank_o_Clostridia_UCG-014* inhibits taurine and TCDCA metabolism, disrupting host cholesterol homeostasis and exacerbating hepatic lipid accumulation.

## CONCLUSION

Overfeeding induces hepatic lipid dysregulation, where reduced *norank_o_Clostridia_UCG-014* abundance diminishes taurine and its conjugated bile acids, triggering lipid droplet accumulation and foie gras formation. The intricate network of overfeeding-associated hepatic lipid metabolism, governed by the gut microbiota-metabolite-liver axis, provides novel insights into the microbiological mechanisms underlying these processes.

## Figures and Tables

**Table 1:** Differentially abundant metabolite traceability analysis

Compounds	CAS	Origin
Taurine	107-35-7	Bacteria, Fungi, others, Archaea, Homo
Tauroursodeoxycholic acid	14605-22-2	Homo
Taurolithocholic acid	516-90-5	Others, Bacteria, Homo
Isolithocholic acid	1534-35-6	Others, Bacteria, Homo
Taurochenodeoxycholic acid	516-35-8	Bacteria, others, Archaea, Homo
Glycolithocholic acid	474-74-8	Others, Bacteria, Homo
